# Seasonal shifts from plant diversity to consumer control of grassland productivity

**DOI:** 10.1111/ele.13993

**Published:** 2022-03-01

**Authors:** Max M. Zaret, Molly A. Kuhs, Jonathan C. Anderson, Eric W. Seabloom, Elizabeth T. Borer, Linda L. Kinkel

**Affiliations:** ^1^ Department of Ecology, Evolution and Behavior University of Minnesota Saint Paul Minnesota USA; ^2^ Department of Plant Pathology University of Minnesota Saint Paul Minnesota USA

**Keywords:** biodiversity‐ecosystem function, fungus, grasslands, insect, plant‐consumer interaction, productivity, vegetation dynamics

## Abstract

Plant biodiversity and consumers are important mediators of energy and carbon fluxes in grasslands, but their effects on within‐season variation of plant biomass production are poorly understood. Here we measure variation in control of plant biomass by consumers and plant diversity throughout the growing season and their impact on plant biomass phenology. To do this, we analysed 5 years of biweekly biomass measures (NDVI) in an experiment manipulating plant species richness and three consumer groups (foliar fungi, soil fungi and arthropods). Positive plant diversity effects on biomass were greatest early in the growing season, whereas the foliar fungicide and insecticide treatments increased biomass most late in the season. Additionally, diverse plots and plots containing foliar fungi reached maximum biomass almost a month earlier than monocultures and plots treated with foliar fungicide, demonstrating the dynamic and interactive roles that biodiversity and consumers play in regulating biomass production through the growing season.

## Graphical abstract

Evidence of plant diversity controls on biomass have historically relied on single time point measurements within the growing season and rarely account for consumer effects on plant biomass. Consequently, we know very little about within‐season variation in these biotic controls. Here, in a grassland diversity and consumer removal experiment, we found that both plant diversity and consumer controls on plant productivity shift in magnitude across the growing season, and diversity and consumers alter the timing of peak grassland biomass. This study offers new evidence that loss of consumer and plant diversity is having previously undocumented impacts on the productivity of terrestrial ecosystems.

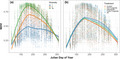

## INTRODUCTION

Plant biomass production provides the energy for most of Earth's terrestrial biological processes, supports a variety of ecosystem services and plays a vital role in regulating climate as human activities alter the global carbon cycle (Balvanera et al., 2006; Beer et al., 2010; Naeem, 1998; Tilman, 2000). Experimental manipulations of species diversity have demonstrated that diverse communities generally have more plant biomass than low diversity communities (Cardinale et al., 2006; Hooper et al., 2005; O’Connor et al., 2017). Additionally, theory and field experiments have demonstrated that diversity effects depend on the composition of the consumer food web (e.g. the abundance of herbivores, pathogens and mutualists, Bruno et al., 2008; Duffy et al., 2007; Schnitzer et al., 2011; Seabloom et al., 2017; Thebault & Loreau, 2003). However, while compelling, diversity‐productivity relationships in terrestrial systems are usually quantified based on one or two harvests of plant biomass, leaving a gap in knowledge about rates of biomass production throughout the growing season. Consumer effects on plant biomass are often characterised multiple times over the growing season (McNaughton et al., 1996), but are rarely measured within biodiversity experiments (Seabloom et al., 2017). The lack of repeated, non‐destructive measurements of plant biomass production through a growing season may bias our understanding of the relative importance of biodiversity and consumer effects on ecosystem function, if these biotic controls on plant productivity vary across a season.

Biodiversity experiments frequently find a positive effect of plant biodiversity on annual peak‐season plant biomass production (standing stock of carbon in plant tissue, Cardinale et al., 2009). This phenomenon is often referred to as an ‘overyielding effect’ in which plant communities with high species richness or functional group diversity exhibit greater annual plant biomass production compared to monocultures (Hooper et al., 2005). While an overyielding effect at peak biomass or at the end of a growing season is a common phenomenon, increased peak season biomass may emerge in a variety of ways. For example, high diversity plant communities may consistently produce more biomass than communities that contain only a few or a single plant species across a growing season, with little variation in the effects of biodiversity on biomass production through the growing season. Alternatively, shifts in the effects of biodiversity on biomass production across a growing season may occur due to factors including seasonal shifts in growth‐limiting resources (Fornara & Tilman, 2009; Hooper, 1998), for example favouring growth of monocultures early but mixtures later in the season. However, measurements at peak season leave a gap in knowledge about within‐season variation in controls on biomass production.

In addition to plant biodiversity, plants directly interact with a broad suite of consumers (heterotrophs) in the food web, such as fungal pathogens, fungal mutualists, arthropod herbivores and plant pollinators. Plant‐consumer interactions and effects of consumers on biomass likely follow seasonal patterns that are constrained by physiological and ecological factors (Ekholm et al., 2020; Walther et al., 2002). For example, arthropods and fungi in temperate regions have seasonal cycles of emergence and growth in response to environmental stimuli including temperature, day length and moisture (Arseniuk et al., 1998; Danilevsky et al., 1970). However, consumer groups vary in composition and impact through a season, such as rapid within‐season turnover in community composition of soil and foliar microbes (Schadt et al., 2003; Voriskova & Baldrian, 2013), variation in plant pathogen phenology (Halliday, Umbanhowar, et al., 2017) and variation in spring emergence times and dietary needs among arthropod herbivores (Danilevsky et al., 1970; Shiojiri & Karban, 2008). Additionally, consumer abundances and impacts on plant communities can vary across gradients of plant diversity, with dilution or amplification of animal and fungal impacts with increasing plant diversity (Borer et al., 2012; Kohli et al., 2021; Mitchell et al., 2002; Rottstock et al., 2014; Seabloom et al., 2017). Despite well‐documented evidence for variation of activity among consumer groups across the growing season and the potential for interactions with plant biodiversity, no studies have yet quantified the within‐season interactions of consumer and plant diversity on plant biomass production. Given declining plant and consumer diversity (Díaz et al., 2019), this gap in knowledge may undermine predictions of impacts on ecosystem functioning.

Phenology, or the timing of biotic events, is key to understanding ecosystem processes (Forrest & Miller‐Rushing, 2010; Wang et al., 2020), and phenology of plant biomass production (timing of maximum plant biomass and rates of biomass green‐up and senescence) can be determined through higher temporal resolution of plant biomass data (Pettorelli et al., 2005). In addition to a lack of studies that quantify within‐season variation in diversity and consumer controls on plant biomass production, few have quantified biotic controls on plant community phenology (Wolf et al., 2017). Yet diversity loss can impact the timing of flowering events and growing season length (Oehri et al., 2017; Wolf et al., 2017), while consumers such as foliar fungi can mediate the timing of leaf senescence (Waggoner & Berger, 1987; Wilson, 1993). However, an integrated understanding of how diversity and consumers interactively control plant biomass phenology remains absent from this field of inquiry.

Here we leverage a long‐term consumer‐removal experiment (removing arthropods, soil fungi, foliar fungi, or concurrent removal of all three trophic groups) nested within a plant biodiversity experiment to investigate how consumers and plant diversity impact within‐season variation in biomass production. To do this, we use five years of biweekly estimates of aboveground plant biomass based on the Normalized Difference Vegetation Index (NDVI) (Running, 1990). In addition to estimating seasonal dynamics of plant biomass, we test the effects of plant diversity and consumers on phenological measures of plant biomass, such as timing of maximum plant biomass and rates of plant biomass green‐up and senescence (Pettorelli et al., 2005). We use these data to ask the following questions:

1) *Do plant diversity and consumer controls on biomass vary across the growing season?* Here we tested a null expectation that plant diversity and consumer impacts on plant biomass production are constant across the season if plant diversity or consumers have proportional effects on plant biomass production (e.g. consumers remove a constant proportion of plant biomass or positive diversity effects on biomass are constant, Seabloom et al., 2017).

2) *Do plant diversity and consumers interact to impact the timing of peak biomass or the rates of plant green*‐*up and senescence?* We predicted that diverse plant communities would have either earlier or later seasonal timings of peak biomass than monocultures due to the combination of species with a variety of temporal niches (e.g. species specific timing of maximum growth). We expected consumers generally not to alter the timing of peak biomass, though necrotrophic fungi have been shown to promote earlier plant senescence (Häffner et al., 2015). Additionally, we predicted faster rates of green‐up and senescence in diverse plant communities and communities with consumers removed if increased biomass (from higher diversity or consumer removal) results from more rapid green‐up due to the larger peak of the biomass time‐series curve (i.e. having steeper slopes of biomass accumulation and senescence as a result of a taller peak biomass value, Petorelli et al. 2005).

## METHODS

### Diversity experiment

We conducted this experiment at the Cedar Creek Ecosystem Science Reserve in East Bethel, Minnesota as part of the U.S Long Term Ecological Research (LTER) Network (Latitude 45.4°N, 93.2°W). The consumer removal experiment, established in 2008, was nested within a long‐term grassland plant diversity experiment started in 1994 (Tilman et al., 2001). Planted richness treatments ranged from 1 to 16 species that were randomly assigned to 168–9 m × 9 m plots, with the composition of each plot being a random subset of 17 native perennial species (C4 grasses, C3 grasses, legumes, and forbs). A list of the plant species and their functional group identity can be found in Table S1. Species richness treatments are maintained through weeding throughout the growing season. The entire experimental field was fenced to exclude deer and other large vertebrate herbivores and is burned after snowmelt every year removing aboveground litter from the previous year.

### Consumer removal experiment

In 2008, the consumer removal experiment treatments were established in 33 of the plant diversity experimental plots, with planted richness of 1 (*n* = 15), 4 (*n* = 9), and 16 species (*n* = 9). Every experimental plot received 5 treatments (control, insecticide, foliar fungicide, soil fungicide, and all pesticides combined) that were randomly assigned to subplots (1 treatment per subplot) of 1.5 × 2 m for a total of 165 experimental subplots (33 diversity plots × 5 treatments) and 15 treatment combinations (three plant species richness treatments, each containing five consumer removal treatments). Subplots were separated by a 0.5 m buffer strip.

### Pesticide treatments

Pesticide treatments were applied regularly throughout the growing season from April to August each year. Foliar fungicide was applied biweekly and composed of Quilt (Syngenta Crop Protection, Inc.), a combination of Azoxystrobin (7.5%) and Propiconazole (12.5%). Soil Fungicide was applied monthly and composed of Ridomil Gold SL (Syngenta Crop Protection, Inc.), a soil drench fungicide containing Mefenoxam (45.3%). Insecticide treatments were applied biweekly and composed of Marathon II (OHP, Inc.), containing Imidacloprid (21.4%). Once or twice each season, Malathion was applied instead of Marathon II to reduce the possibility of insecticide resistance by the local insect populations.

Previous work from this experiment has shown that these pesticide treatments significantly reduce foliar damage by insect herbivores and foliar pathogens (Borer et al., 2015) and results from a greenhouse experiment showed that none of the treatments impact plant biomass in the absence of consumers (Seabloom et al., 2017). We did not perform DNA sequencing or insect collection to determine how specific fungi, or arthropod taxa are impacted by our pesticide treatments. However, Mitchell et al. (2002) found that at this site, our focal plant species are most commonly infected by *Colletotrichum sp*. (fungal leaf spot), *Erysiphe cichoracearum* (powdery mildew) and *Uromyces sp*. (fungal rust), all of which are targets of our foliar fungicide. Additionally, these experimental plots have been shown to host a broad array of arthropods, representing 13 insect orders, with herbivores representing 62% of the taxa collected (Borer et al., 2012). Finally, the pesticides used in this study may not be effective on all the taxa in each consumer group (e.g. the soil drench fungicide may only impact oomycetes without impacting other soil fungi) so our food web manipulations and results represent conservative estimates of the impact of consumer groups on plant biomass.

### NDVI data

For 8 years (2009–2016), we used a MSR5 multispectral radiometer (Cropscan, Inc.) to measure reflected radiation (reflectance) in all experimental plots. Measurements were taken every two weeks from April to August in a 1.5 m^2^ area in the center of each plot. Normalized Difference Vegetation Index (NDVI) was derived from the red: near‐infrared reflectance ratio (Running, 1990) using 830 mm (near‐infrared) and 660 mm (red) reflectance readings. NDVI values were calculated for each plot on each day of measurement resulting in 20,135 NDVI estimates across the entire study.

### NDVI time‐series metrics and calculations

A variety of ecologically relevant metrics of NDVI time series data were calculated in R version 4.0.0 (R Core Team 2013) to determine the impacts of plant diversity and food web composition on primary production. Integrated NDVI (INDVI) is the sum of NDVI values over a given time period and is a proxy for overall plant production (e.g. biomass, Pettorelli et al., 2005). INDVI was calculated as the numerical integral under a smoothing spline interpolation of NDVI values using the ‘MESS’ package and was partitioned into three metrics of INDVI: INDVI for the entire growing season (including all NDVI values from the time‐series of a plot in a given year), early season INDVI (first 50% of NDVI values), and late season INDVI (last 50% of NDVI values of the time series) to look at variation in INDVI responses across the growing season. We note here that results were comparable when we divided the growing season by day of peak NDVI rather than median day of year. Three years of data (2009, 2012, 2015) were excluded from INDVI calculations due to poor sampling during the early or late season (i.e. we were unable to characterise the entire growing season due to a lack of measurements from early or late season dates). In total, 825 smoothing splines were made to calculate INDVI, and the average *R* ^2^ value for the NDVI smoothing splines was 0.91, with a minimum value of 0.28 and a maximum of 0.99. INDVI for the entire growing season correlated with aboveground biomass estimates (Figure S1., *R* ^2^ = 0.36, *p* < 0.0001).

Phenological measures also can be derived from NDVI time‐series data (Pettorelli et al., 2005). We estimated rates of NDVI accumulation (green‐up) by fitting linear functions to all NDVI values that occur on the day of year for a given experimental plot before maximum (peak) NDVI, producing the slope of NDVI values against time (day of year). We used the same approach to look at NDVI declines (senescence) by using all NDVI values that occur on the day of year after peak NDVI. Experimental plots exhibited unimodal trends in NDVI (one distinct peak in NDVI across the growing season) and thus partitioning NDVI values by pre or post peak NDVI produced positive slopes for NDVI green‐up estimates and negative slopes for estimates of NDVI senescence. The average *R* ^2^ values for the NDVI green‐up and senescence linear regressions was 0.82. Finally, the timing of peak NDVI represents an important metric for quantifying the temporal dynamics of energetic subsidies via carbon to the food web. We defined timing of peak NDVI as the Julian day of year when the maximum NDVI value occurs within a given year within a given experimental plot.

### Statistical analysis

We used linear mixed‐effect models to determine the effects of plant diversity and consumer removal treatments on various NDVI time‐series metrics (INDVI, rates of NDVI accumulation/decline, and timing of peak NDVI) using the lme function in the nlme package (Pinheiro et al., 2017). Plant diversity and consumer removal treatments were included as fixed effects, while sampling year, the 9 × 9 m plant diversity plots and the 1.5 × 2 m subplots were treated as random effects to account for the nested study design. *R* ^2^ values from the smoothing splines and linear regressions used to derive INDVI, rate of NDVI accumulation, and rate of NDVI decline metrics were incorporated into models as weights (using weights function in lme), where experimental plots with higher *R* ^2^ values have more weight than plots with lower *R* ^2^ values. To ensure there was no effect of temporal autocorrelation, we included sampling year as a correlation term in the mixed effect models, but this did not improve models (based on a likelihood ratio test). Finally, we used the lsmeans package to compute least‐square means to report effect sizes as well as differences among plant diversity and consumer removal treatments (reporting standard error of each least‐square means).

## RESULTS

**FIGURE 1:**
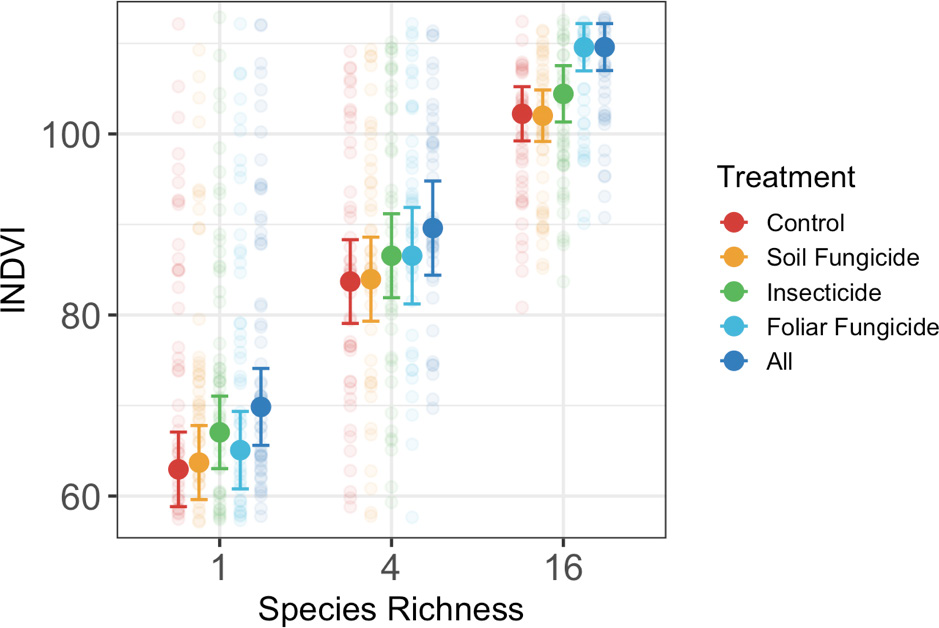
Comparison of plant and trophic diversity effects on INDVI across entire growing season. When looking at whole growing season, there are strong plant and consumer group effects, as well as interactions for both insects and foliar fungi. INDVI is the integrated NDVI values for a given time period (whole season here). Solid points represent mean INDVI values for a given treatment and bars represent 95% confidence intervals

When integrated across the growing season, plant diversity increased INDVI (Table S2; Figure 1), with 16 species plots having 60.7% (SE = 5.68) greater INDVI values compared to monocultures (SE =standard error). All consumer removal treatments except soil fungicide increased INDVI on average when looking across the growing season. The all‐pesticides treatment had the strongest effect, increasing INDVI by 8.1% (SE = 5.93). Interactions between plant diversity and consumer removal occurred when looking at data integrated across the growing season. In particular, removal of foliar fungi increased INDVI by 7.2% (SE = 1.14) in 16 species plots but had no effect in monoculture.

**FIGURE 2:**
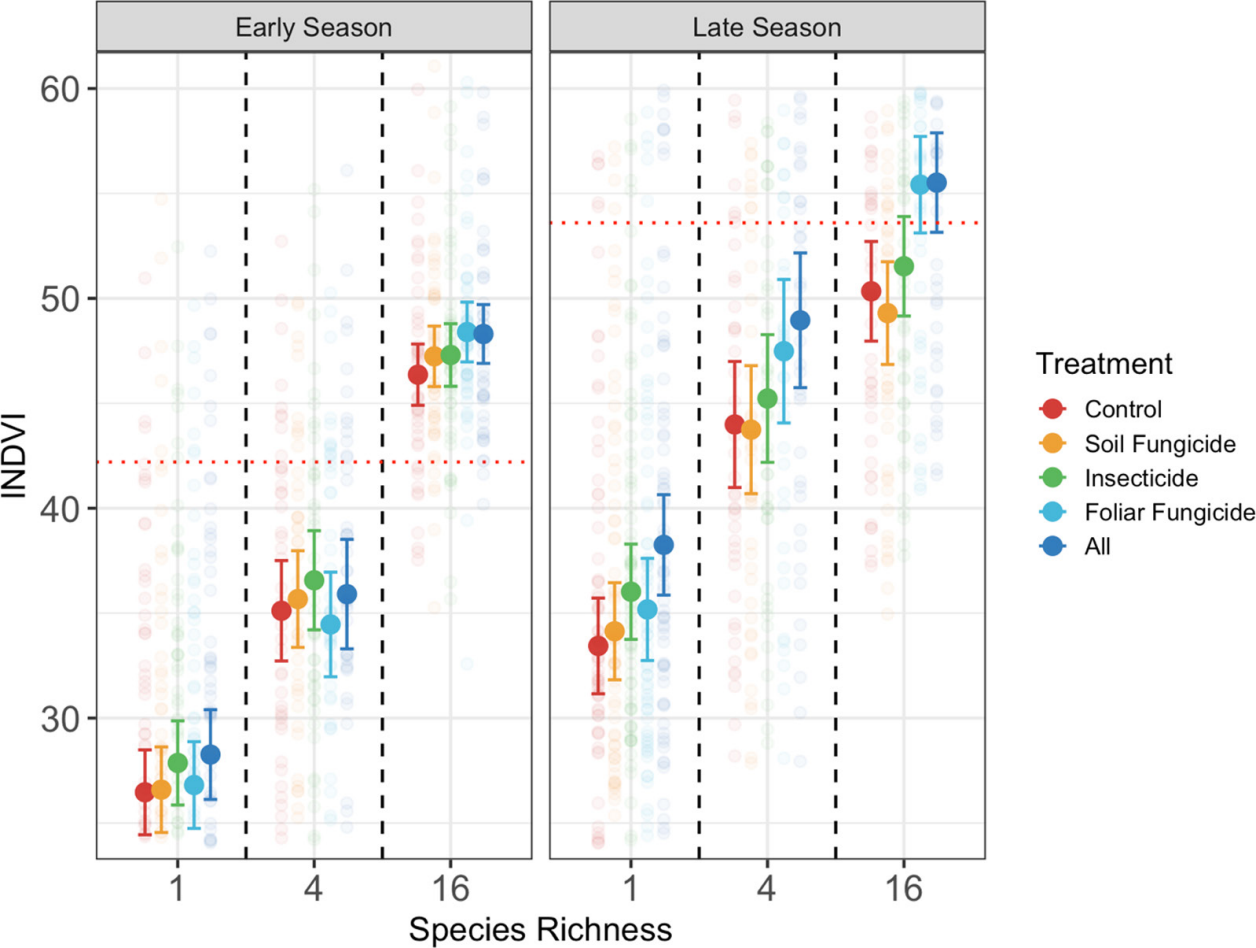
The impacts of plant diversity, consumers, and their interaction on productivity vary across the growing season. Early season integrates the first 50% of seasonal NDVI values while late season integrates the remaining 50% of seasonal NDVI values. Solid points represent mean INDVI values for a given treatment and bars represent 95% confidence intervals. Red dotted line represents INDVI of most productive monoculture in the early or late season

Plant diversity had the strongest effects on INDVI early in the growing season (Table S3; Figure 2), where 16 species plots had 74.6% greater INDVI (SE = 1.33) compared to monocultures. Consumer removal effects were also significant, but relatively small, early in the growing season and were driven by the insecticide treatment increasing INDVI by 3% (SE = 0.406). There was also an interaction between plant diversity and foliar fungi removal treatments for early INDVI: foliar fungicide application increased early INDVI by 3.6% (SE = 0.607) in 16 species plots but did not impact early INDVI in monocultures.

Late in the growing season, 16 species plots had 48% (SE = 1.42) higher INDVI than monocultures (Table S4; Figure 2). In contrast to the early season, all consumer removal treatments except the soil fungicide increased INDVI, and consumer removal treatments interacted with plant diversity. The foliar fungicide treatment increased INDVI by 10% (SE = 0.949) in 16 species plots but did not consistently impact INDVI in monocultures. In contrast, the insecticide treatment increased INDVI by 8% (SE = 0.735) in monocultures but had no significant effect in 16 species plots. The soil fungicide treatment also significantly interacted with diversity, but effects were small with the removal of soil fungi reducing late season INDVI by 1.8% (SE = 0.631) in 16 species plots. The all‐pesticides treatment was stronger than any individual pesticide application, increasing INDVI by 12% (SE = 0.339) across all diversity treatments.

**FIGURE 3:**
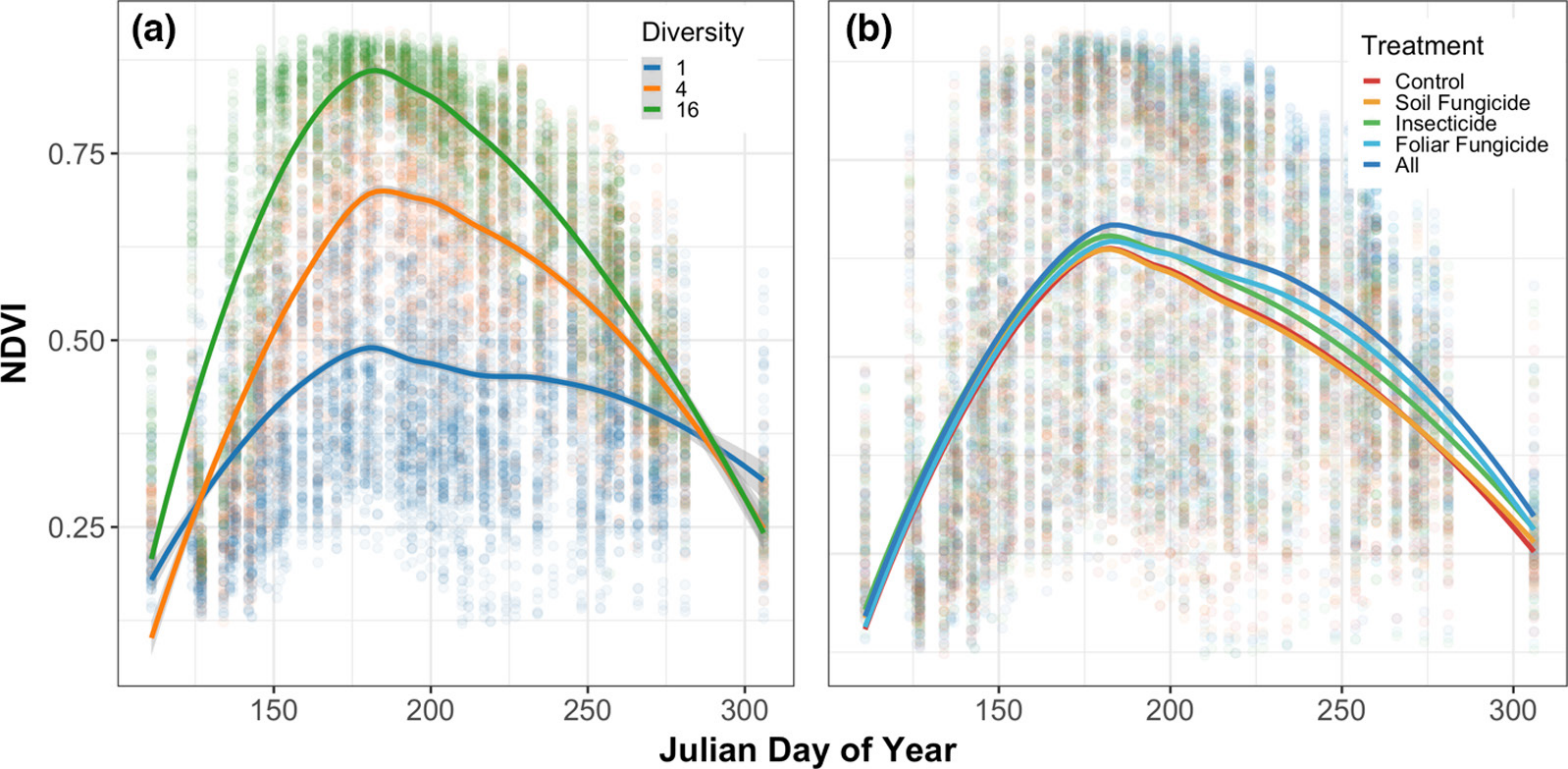
Plant diversity influences rates of vegetation green‐up (accumulation) and senescence (a), while consumers impact vegetation senescence (b). Trend lines show local polynomial regression fit of each experimental treatment with an alpha parameter of 0.75

**FIGURE 4:**
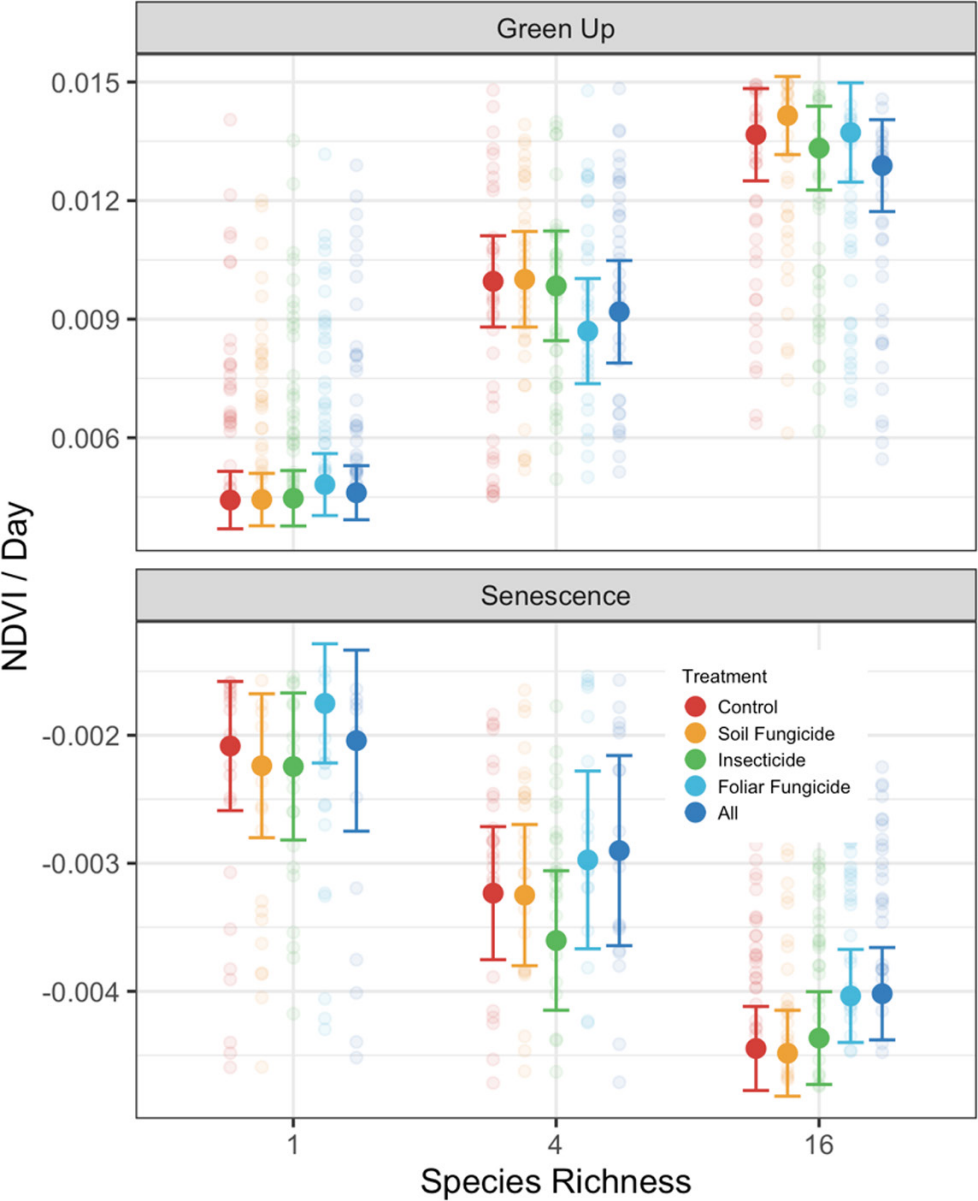
Slope estimates of seasonal NDVI accumulation (Green Up) and decline (Senescence) across plant diversity and consumer removal treatments. Solid points represent mean slope values for a given treatment and bars represent 95% confidence intervals

During the period of vegetation green‐up, plant diversity increased rates of NDVI accumulation (Table S5; Figure 3a), with NDVI in 16 species plots increasing three times faster than in monocultures. During the early season period, there was no effect of consumer removal on rates of NDVI accumulation (Figure 3b), but there was an interaction between plant diversity and the foliar fungicide treatment (Figure 4) in which rates of green‐up in 4 species plots were reduced by 14.4% (SE = 0.0008).

Rates of NDVI decline varied with plant diversity (Table S6, Figure 3a), with NDVI values in 16 species plots decreasing eight times faster than monocultures. All consumer removal treatments slowed rates of NDVI decline (Figure 3b) and interacted with plant diversity to influence rates of senescence (Figure 4). Delayed senescence in the foliar fungicide treatment was greatest in 4 species plots where senescence was 35.7% (SE = 0.0001) slower than control plots.

**FIGURE 5:**
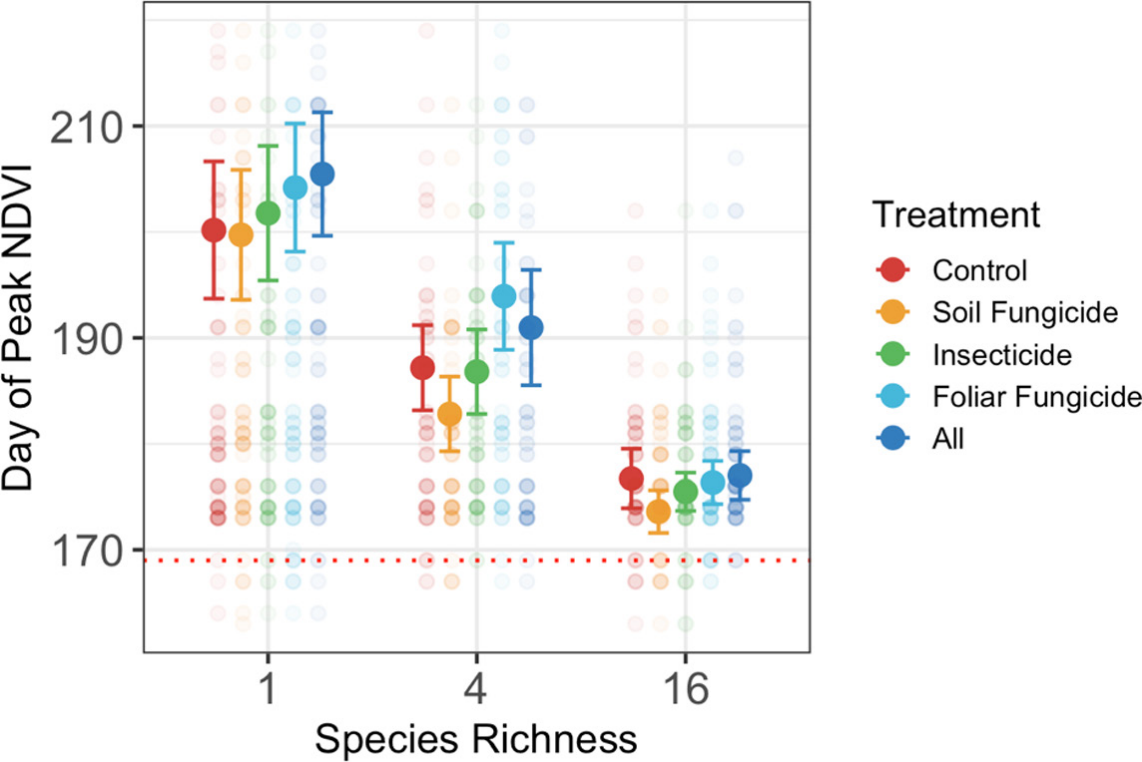
Plant diversity and consumer group impacts on timing of the maximum NDVI value for a given plot during a growing season, or peak NDVI. Loss of plant diversity and loss of aboveground consumers cause peak NDVI to occur later in the growing season than control conditions. Solid points represent mean Julian day of peak NDVI values for a given treatment and bars represent 95% confidence intervals. Red dotted line represents average day of peak NDVI of the earliest peaking monoculture

Finally, timing of peak NDVI was significantly altered by plant diversity and consumer removal treatments, but these factors acted independently (Table S7; Figure 5). The 16 species plots reached peak NDVI 26 days (SE = 6.41) earlier than monocultures on average. Day of peak NDVI and NDVI curves of monocultures in general were highly variable among plant species (Figure S2), with some species peaking earlier than 16 species plots (*Lupinus perennis* peaked in early June on average across years) while other species peaked late in the growing season (*Achillea millefolium* peaked in late September on average across years). Treatment plots containing foliar fungicide (foliar fungicide and all‐pesticides) peaked 3 days (SE = 1.81) later than controls.

## DISCUSSION

We found strong seasonal differences in both plant diversity and consumer group impacts on biomass production. Specifically, early season productivity is more strongly controlled by plant diversity, whereas consumers become more important in controlling later season productivity. In general, interactions between plant diversity and consumer groups were weak earlier in the growing season but became stronger as the growing season progressed. Additionally, both plant diversity and consumers altered timing of maximum NDVI and rates of green‐up and senescence. Diverse plant communities had faster green‐up of NDVI and reached peak NDVI almost a month earlier than monocultures. Consumers more strongly impacted NDVI senescence, with the foliar fungicide substantially slowing senescence and delaying the timing of maximum NDVI in the community. These results highlight the important—and shifting—roles of both plant diversity and consumer groups on productivity over the growing season and offer new insights into how carbon cycling may be altered in response to biodiversity loss and simplification of food webs in disturbed terrestrial ecosystems.

Our results demonstrate that the positive plant diversity effects on plant biomass production decline through the growing season. In particular, the strong increase in NDVI with increasing plant diversity early in the growing season generated dramatic differences between the high diversity plots compared to four species plots and monocultures. In contrast to these strong, positive early season effects, after peak NDVI the high diversity plots exhibited rapid declines with NDVI decreasing eight times faster compared to monoculture. These results suggest that positive diversity‐productivity relationships can be dynamic through a growing season, potentially due to shifts in complementarity effects (Hooper, 1998) and shifts in growth limiting resources such as soil nutrients or water content (Fornara & Tilman, 2009). Specifically, the presence of multiple functional groups in a community (C4 grasses, C3 grasses, and forbs) can deplete soil N (Fornara & Tilman, 2009) and soil water (Verheyen et al., 2008) earlier in the season than monocultures. Although not measured in this study, more complete use of resources in the early season by high diversity communities may explain the contrasting early versus late season effects and explain the more rapid rates of decline later in the season if nutrient or water demands far outpace the available supply by the mid to late season. More broadly, this work indicates that studies investigating biodiversity–productivity relationships in temperate grasslands may under or overestimate diversity effects if biomass measurements are only taken one or two times during the growing season. It will be important to compare the seasonal variation in diversity–productivity relationships observed in this experimental plant community (where diversity is maintained through weeding) to those in unmanaged, natural systems that likely have more complex feedbacks between plant species richness and biomass production (Wardle, 2016). Given that plant production provides the energy to all organisms in food webs, these insights will be especially important to predict how biodiversity loss or gain will alter local plant carbon fluxes to the food web through a growing season.

Both arthropods and foliar fungi predominantly impacted plant biomass production late in the growing season. These findings support the concept of trophic control by plant consumers in temperate regions being dependent on the seasonal timing of plant emergence, growth and senescence (Ekholm et al., 2020). While the soil fungicide treatment did not alter NDVI in this study, past work in this system has found that plant species are impacted by the removal of soil fungi. In particular, forb species tend to increase in biomass with the removal of soil fungi (Seabloom et al., 2018). The lack of NDVI response also may be due to the soil fungicide impacting harmful pathogens and beneficial mutualists, resulting in no net change in biomass. Alternatively, some pathogens unaffected by soil fungicide may compensate when others are removed. Other consumer groups differed in their temporal variation of consumer control on biomass, with arthropods having significant impacts earlier in the season compared to foliar fungi. Such seasonal differences in arthropod and fungal control of plant biomass production may be due to the high mobility of arthropods that are able to disperse into the system early or emerge from overwintering faster (Jonsen & Fahrig, 1997), while fungal impacts on plant biomass may build more slowly via local growth from spores that accumulate and mature over the growing season (which is more likely for biotrophs than nectrophs, Money, 2016; Précigout et al., 2020). Although variable, interactions between consumers and plant diversity tended to increase through the growing season. In contrast to the earlier season arthropod impacts, the late season impacts of foliar fungi were greatest among all the consumer groups tested in this study, adding to previous work that highlights the potential importance of aboveground fungi as mediators of ecosystem processes such as plant biomass production (Allan et al., 2010; Kohli et al., 2021; Mitchell, 2003; Preston et al., 2016; Seabloom et al., 2017).

Impacts on plant biomass across plant diversity also differed by consumer group. Specifically, arthropod impacts on biomass accumulation were greatest at low diversity, whereas foliar fungi had the largest effects in the highest diversity plots. Interestingly, these impacts are broadly concordant with impacts of these consumer groups on foliar percent nitrogen (Borer et al., 2015), suggesting that plant diversity may mediate links between consumers and plant physiology. Previous studies have found substantial and interactive effects of plant diversity and consumers on harvested peak season biomass (Seabloom et al., 2017) and rates of gross primary production (Kohli et al., 2021). Our results build from these to demonstrate that foliar fungi and arthropods have differing impacts on plant biomass across a gradient of plant diversity and influence within‐season patterns of plant biomass accumulation and loss. These findings also suggest that generalist fungi in grasslands may be stronger drivers of plant production compared to host‐specialists, while the opposite may be true for arthropod herbivores that may be better able to seek out specific plant hosts across larger spatial scales (biodiversity plots) compared to fungi (Jonsen & Fahrig, 1997). One explanation for amplified fungal effects on biomass at high diversity is that diverse communities may be more likely to contain a highly susceptible plant host that serves as a reservoir for disease and greatly increases pathogen prevalence in other species if the pathogen is a generalist (pathogen spillover, Power & Mitchell, 2004) or amplification may be due to an increase in pathogens that do not respond to host density (such as vector transmitted pathogens, Halliday, Heckman, et al., 2017). Epidemiological factors that may vary with plant diversity, including foliar density and changes in microclimate (e.g. relative humidity), as well as seasonal variation in plant chemistry, resistance or fungal inoculum are also likely to play important roles in mediating the impacts of foliar fungi on plant biomass (Elad & Pertot, 2014; Häffner et al., 2015; Huber & Gillespie, 1992). Overall, these findings demonstrate that plant diversity interacts with the composition of food webs (identity of consumer groups) to determine patterns of plant biomass production (Duffy et al., 2007).

Our work clearly demonstrates that both plant diversity and consumers play a key role in plant phenology. In particular, increased plant diversity accelerated plant biomass green‐up and caused the timing of peak productivity and plant biomass senescence to shift earlier in the season. Previous work quantifying phenology shifts of individual species across gradients of plant diversity found that increasing plant diversity led to later leaf‐out and flowering events (Du et al., 2019; Wolf et al., 2017), yet these findings are not necessarily at odds with our work. Rather, this highlights variation in phenology of individual species versus aggregate characteristics of entire plant communities (biomass or NDVI) in response to diversity. The two plant species in this study system with the earliest day of peak NDVI (*Lupinus perennis* and *Liatris aspera*) had peak days in monoculture that were earlier than the peak day of 16 species plots (Figure S2, Figure 5), suggesting that the earlier peak biomass in 16 species plots may result from the presence of these early seasons in mixtures. However, when we compared the most productive monoculture to 16 species plots in the early season versus late season, the early season high diversity plots had higher INDVI than the most productive monoculture, but this overyielding effect decreased later in the season (Figure 2). This may indicate that the increased early season biomass production is more likely a function of complementarity effects (however such calculations require species specific biomass in mixture which we could not measure in the early season). As with plant diversity, consumer impacts on the timing of peak productivity also altered phenology. Foliar fungicide, in particular, slowed rates of senescence and delayed the time to maximum NDVI, suggesting the importance of this consumer group in speeding plant senescence (Waggoner & Berger, 1987; Wilson, 1993). While the fungicide, rather than the fungi, may be implicated, here, a greenhouse study found no differences in biomass induced by these treatments (Seabloom et al., 2017), suggesting that the fungi, themselves, are likely inducing this response. Earlier work demonstrated that diversity of the biotic community can also influence growing season length (Oehri et al., 2017). However, our study builds from this earlier work to experimentally quantify the importance and interactions of plant diversity and consumers on the patterns of within‐season biomass phenology that are critical to understanding the dynamics of carbon provisions to food webs and ecosystems (Pettorelli et al., 2005; Wang et al., 2020).

Overall, our findings demonstrate that plant diversity, consumers, and their interactions impose significant controls on grassland plant biomass production that shift in intensity through the growing season. Plant diversity speeds phenology, with faster early season growth and senescence occurring nearly a month earlier in high diversity plant communities. Foliar fungi also speed senescence. In addition, diversity effects on plant biomass may be significantly greater than previously estimated, particularly via controlling the rate of early season biomass accumulation. Plant diversity also shapes the impact of different consumer groups on biomass accumulation, peak timing and senescence, with greater impacts of arthropods at low diversity and fungi at high diversity. Additionally, our results suggest that ongoing plant and trophic diversity loss may push timing of maximum productivity later in the growing season, potentially impacting animal or microbial populations that rely on vegetation phenology but respond to different phenological cues. These new insights into the roles of plant diversity and consumers on the temporal dynamics of temperate grassland vegetation will contribute to more effective predictions of carbon uptake and timing of carbon subsidies to food webs with ongoing biodiversity loss.

## AUTHORSHIP

EB, ES and LK coordinated the consumer removal experiment. MZ, MK, ES and LK conceived this study. JA collected data. MZ analysed data with inputs from MK, ES and LK. MZ wrote the first draft of the manuscript. All authors contributed substantially to manuscript revisions.

### PEER REVIEW

The peer review history for this article is available at https://publons.com/publon/10.1111/ele.13993.

## Supporting information

Supplementary Material

## Data Availability

Data are deposited at the Environmental Data Initiative (https://doi.org/10.6073/pasta/e1b55756a34c63cd949d44a5bad9f601), and code to reproduce the analysis is deposited at Zenodo (https://doi.org/10.5281/zenodo.6025528).
