# Supplementary material for: Seasonal shifts from plant diversity to consumer control of grassland productivity
Source: Ecol Lett. 2022 Mar 1;25(5):1215–24. doi: 10.1111/ele.13993 (PMC9544143; doi:10.1111/ele.13993)
Supplement: Supplementary file 1 — Supplementary Material [file ELE-25-1215-s001.docx]

**Supplementary Information for**

Seasonal shifts from plant diversity to consumer control of grassland productivity

Max M. Zaret^1*^, Molly A. Kuhs^1^, Jonathan C. Anderson^2^, Eric W. Seabloom^1^, Elizabeth T. Borer^1^, Linda L. Kinkel^2^

1. Department of Ecology, Evolution and Behavior, University of Minnesota, Saint Paul, MN
2. Department of Plant Pathology, University of Minnesota, Saint Paul, MN

^*^**Author for Correspondence:** Max M. Zaret

*Address: Department of Ecology, Evolution and Behavior, University of Minnesota, 1479 Gortner Ave, Saint Paul, MN 55108; Email:* [*zaret007@umn.edu*](mailto:zaret007@umn.edu)

**This PDF file includes:**

Tables S1 to S7

Figures S1 to S2

**Supplemental Tables.**

**Table S1.** Plant species included in the plant diversity experiment and their associated plant functional group. In mixtures (4 or 16 species plots), the composition of each plot was a random subset of these 19 native perennial species.

**Table S2.** Whole growing season (annual) INDVI response to consumer removal treatments (soil fungicide, insecticide, foliar fungicide, and all pesticides combined) across a gradient of plant species richness (1, 4, and 16 species). This table is linked with Figure 1 in the main text.

$$Annual INDVI \sim Diversity*Removal Treatment, random=\sim1 | Year/Plot/Subplot$$

| **Source** | **numDF** | **denDF** | **F-value** | **p-value** |  |  |  |  |  |  |  |  |
| --- | --- | --- | --- | --- | --- | --- | --- | --- | --- | --- | --- | --- |
| **(Intercept)** | **1** | **648** | **849.2927** | **<.0001** |  |  |  |  |  |  |  |  |
| **Diversity** | **2** | **158** | **110.6354** | **<.0001** |  |  |  |  |  |  |  |  |
| **Removal Treatment** | **4** | **648** | **45.1506** | **<.0001** |  |  |  |  |  |  |  |  |
| **Diversity: Removal Treatment** | **8** | **648** | **3.7664** | **0.0002** |  |  |  |  |  |  |  |  |
|  |  |  |  |  |  |  |  |  |  |  |  |  |
| **Full Model:**  **Entire Growing Season INDVI** |  |  |  |  |  |  |  |  |  |  |  |  |
|  |  |  |  |  |  |  |  |  |  |  |  |  |
| **Parameter** | **Estimate** | **Standard Error** | **DF** | **t** | **p** |  |  |  |  |  |  |  |
| **(Intercept)** | **63.19** | **3.13** | **648** | **20.20** | **<0.0001** |  |  |  |  |  |  |  |
| **Diversity 4** | **20.51** | **2.84** | **158** | **7.21** | **<0.0001** |  |  |  |  |  |  |  |
| **Diversity 16** | **3902** | **2.84** | **158** | **13.72** | **<0.0001** |  |  |  |  |  |  |  |
| Soil Fungicide | 0.33 | 0.90 | 648 | 0.36 | 0.7123 |  |  |  |  |  |  |  |
| **Insecticide** | **3.75** | **0.90** | **648** | **4.15** | **<0.0001** |  |  |  |  |  |  |  |
| **Foliar Fungicide** | **2.07** | **0.90** | **648** | **2.39** | **0.0214** |  |  |  |  |  |  |  |
| **All Pesticides** | **6.72** | **0.90** | **648** | **7.50** | **<0.0001** |  |  |  |  |  |  |  |
| Diversity 4: Soil Fungicide | -0.03 | 1.42 | 648 | -0.02 | 0.9831 |  |  |  |  |  |  |  |
| Diversity 16: Soil Fungicide | -0.52 | 1.41 | 648 | -0.37 | 0.7110 |  |  |  |  |  |  |  |
| Diversity 4: Insecticide | -1.22 | 1.42 | 648 | -0.65 | 0.5149 |  |  |  |  |  |  |  |
| Diversity 16: Insecticide | -1.66 | 1.41 | 648 | -1.1 | 0.2699 |  |  |  |  |  |  |  |
| Diversity 4: Foliar Fungicide | 0.74 | 1.42 | 648 | 0.62 | 0.5326 |  |  |  |  |  |  |  |
| **Diversity 16: Foliar Fungicide** | **5.27** | **1.41** | **648** | **3.73** | **0.0002** |  |  |  |  |  |  |  |
| Diversity 4: All Pesticides | -0.82 | 1.42 | 648 | -0.59 | 0.5584 |  |  |  |  |  |  |  |
| Diversity 16: All Pesticides | 0.64 | 1.41 | 648 | 0.46 | 0.6468 |  |  |  |  |  |  |  |

**Table S3.** Early season INDVI response to consumer removal treatments (soil fungicide, insecticide, foliar fungicide, and all pesticides combined) across a gradient of plant species richness (1, 4, and 16 species). Early season is defined as first 50% of NDVI values from each plot in a given year. This table is linked with Figure 2A in the main text.

$$Early INDVI \sim Diversity*Removal Treatment, random=\sim1 | Year/Plot/Subplot$$

| **Source** | **numDF** | **denDF** | **F-value** | | **p-value** |  |
| --- | --- | --- | --- | --- | --- | --- |
| **(Intercept)** | **1** | **648** | **584.2077** | | **<.0001** |  |
| **Diversity** | **2** | **158** | **117.5117** | | **<.0001** |  |
| **Removal Treatment** | **4** | **648** | **8.0118** | | **<.0001** |  |
| **Diversity: Removal Treatment** | **8** | **648** | **2.6737** | | **0.016** |  |
|  |  |  |  | |  |  |
| **Full Model:  Early Season INDVI** |  |  |  | |  |  |
|  |  |  | |  |  |  |
| **Parameter** | **Estimate** | **Standard Error** | **DF** | | **t** | **p** |
| **(Intercept)** | **26.46** | **1.6** | **648** | | **16.63** | **<0.0001** |
| **Diversity 4** | **8.75** | **1.41** | **158** | | **6.1** | **<0.0001** |
| **Diversity 16** | **20.02** | **1.41** | **158** | | **14.09** | **<0.0001** |
| Soil Fungicide | 0.12 | 0.47 | 648 | | 0.07 | 0.9447 |
| **Insecticide** | **1.37** | **0.47** | **648** | | **2.75** | **0.0062** |
| Foliar Fungicide | 0.35 | 0.47 | 648 | | 0.69 | 0.4925 |
| **All Pesticides** | **1.8** | **0.47** | **648** | | **3.69** | **0.0002** |
| Diversity 4: Soil Fungicide | 0.34 | 0.75 | 648 | | 0.7 | 0.4854 |
| Diversity 16: Soil Fungicide | 0.63 | 0.75 | 648 | | 1.13 | 0.2609 |
| Diversity 4: Insecticide | -0.12 | 0.75 | 648 | | 0.23 | 0.8206 |
| Diversity 16: Insecticide | -0.44 | 0.75 | 648 | | -0.46 | 0.6489 |
| Diversity 4: Foliar Fungicide | -1.1 | 0.75 | 648 | | -1.28 | 0.2015 |
| **Diversity 16: Foliar Fungicide** | **1.57** | **0.75** | **648** | | **2.3** | **0.0223** |
| Diversity 4: All Pesticides | -1.21 | 0.75 | 648 | | -1.26 | 0.2116 |
| Diversity 16: All Pesticides | -0.13 | 0.75 | 648 | | 0.31 | 0.7636 |

**Table S4.** Late season INDVI response to consumer removal treatments (soil fungicide, insecticide, foliar fungicide, and all pesticides combined) across a gradient of plant species richness (1, 4, and 16 species). Late season is defined as last 50% of NDVI values from each plot in a given year. This table is linked with Figure 2B in the main text.

$$Late INDVI \sim Diversity*Removal Treatment, random=\sim1 | Year/Plot/Subplot$$

| **Source** | **numDF** | **denDF** | **F-value** | **p-value** |  |
| --- | --- | --- | --- | --- | --- |
| **(Intercept)** | **1** | **648** | **207.7109** | **<.0001** |  |
| **Diversity** | **2** | **158** | **76.8924** | **<.0001** |  |
| **Removal Treatment** | **4** | **648** | **86.4056** | **<.0001** |  |
| **Diversity: Removal Treatment** | **8** | **648** | **6.7587** | **<.0001** |  |
|  |  |  |  |  |  |
| **Full Model:**  **Late Season INDVI** |  |  |  |  |  |
|  |  |  |  |  |  |
| **Parameter** | **Estimate** | **Standard Error** | **DF** | **t** | **p** |
| **(Intercept)** | **33.48** | **3.07** | **648** | **10.88** | **<0.0001** |
| **Diversity 4** | **10.53** | **1.51** | **158** | **6.94** | **<0.0001** |
| **Diversity 16** | **16.87** | **1.51** | **158** | **11.14** | **<0.0001** |
| Soil Fungicide | 0.63 | 0.49 | 648 | 1.43 | 0.1994 |
| **Insecticide** | 2.58 | **0.5** | **648** | **5.32** | **<0.0001** |
| **Foliar Fungicide** | **1.77** | **0.49** | **648** | **3.56** | **0.0003** |
| **All Pesticides** | **4.79** | **0.49** | **648** | **9.85** | **<0.0001** |
| Diversity 4: Soil Fungicide | -0.87 | 0.79 | 648 | -1.06 | 0.2724 |
| **Diversity 16: Soil Fungicide** | **-1.67** | **0.79** | **648** | **-2.03** | **0.0351** |
| Diversity 4: Insecticide | -1.34 | 0.8 | 648 | -1.46 | 0.0921 |
| Diversity 16: Insecticide | -1.38 | 0.79 | 648 | -1.76 | 0.082 |
| **Diversity 4: Foliar Fungicide** | **1.77** | **0.8** | **648** | **2.33** | **0.0256** |
| **Diversity 16: Foliar Fungicide** | **3.32** | **0.79** | **648** | **4.33** | **<0.0001** |
| Diversity 4: All Pesticides | 0.18 | 0.8 | 648 | 0.43 | 0.8284 |
| Diversity 16: All Pesticides | 0.4 | 0.79 | 648 | 0.73 | 0.6165 |

**Table S5.** Rate of NDVI accumulation (green-up) response to consumer removal treatments (soil fungicide, insecticide, foliar fungicide, and all pesticides combined) across a gradient of plant species richness (1, 4, and 16 species). Rates of NDVI accumulation are estimated by fitting linear functions to all NDVI values that occur on the day of year before maximum (peak) NDVI, producing the slope of NDVI values against time (day of year). This table is linked with Figure 3 and Figure 4 in the main text.

$$NDVI green up \sim Diversity*Removal Treatment, random=\sim1 | Year/Plot/Subplot$$

| **Source** | **numDF** | **denDF** | **F-value** | **p-value** |  |
| --- | --- | --- | --- | --- | --- |
| **(Intercept)** | **1** | **648** | **104.049** | **<.0001** |  |
| **Diversity** | **2** | **158** | **126.297** | **<.0001** |  |
| Removal Treatment | 4 | 648 | 1.448 | 0.2164 |  |
| **Diversity: Removal Treatment** | **8** | **648** | **2.349** | **0.0171** |  |
|  |  |  |  |  |  |
| **Full Model:**  **Rates of NDVI Green-up** |  |  |  |  |  |
|  |  |  |  |  |  |
| **Parameter** | **Estimate** | **Standard Error** | **DF** | **t** | **p** |
| **(Intercept)** | **0.0044** | **0.0008** | **648** | **5.5** | **<0.0001** |
| **Diversity 4** | **0.0056** | **0.0007** | **158** | **8.59** | **<0.0001** |
| **Diversity 16** | **0.0093** | **0.0007** | **158** | **14.3** | **<0.0001** |
| Soil Fungicide | 0.0002 | 0.0004 | 648 | 0.31 | 0.7586 |
| Insecticide | 0.0002 | 0.0004 | 648 | 0.34 | 0.7359 |
| Foliar Fungicide | 0.0005 | 0.0004 | 648 | 1.32 | 0.1884 |
| **All Pesticides** | **0.0003** | **0.0004** | **648** | **0.72** | **0.4773** |
| Diversity 4: Soil Fungicide | -0.0001 | 0.0006 | 648 | -0.1 | 0.9266 |
| Diversity 16: Soil Fungicide | 0.0004 | 0.0006 | 648 | 0.7 | 0.4892 |
| Diversity 4: Insecticide | -0.0003 | 0.0006 | 648 | -0.42 | 0.6791 |
| Diversity 16: Insecticide | -0.0005 | 0.0006 | 648 | -0.83 | 0.4084 |
| **Diversity 4: Foliar Fungicide** | **-0.0018** | **0.0006** | **648** | **-3.11** | **0.002** |
| Diversity 16: Foliar Fungicide | -0.0004 | 0.0006 | 648 | -0.72 | 0.4764 |
| Diversity 4: All Pesticides | -0.0011 | 0.0006 | 648 | -1.85 | 0.0656 |
| **Diversity 16: All Pesticides** | **-0.0011** | **0.0006** | **648** | **-1.87** | **0.0621** |

**Table S6.** Rate of NDVI decline (senescence) response to consumer removal treatments (soil fungicide, insecticide, foliar fungicide, and all pesticides combined) across a gradient of plant species richness (1, 4, and 16 species). Rates of NDVI accumulation are estimated by fitting linear functions to all NDVI values that occur on the day of year after maximum (peak) NDVI, producing the slope of NDVI values against time (day of year). This table is linked with Figure 3 and Figure 4 in the main text.

$$NDVI senescence \sim Diversity*Removal Treatment, random=\sim1 | Year/Plot/Subplot$$

| **Source** | **numDF** | **denDF** | **F-value** | **p-value** |  |
| --- | --- | --- | --- | --- | --- |
| **(Intercept)** | **1** | **648** | **18.65679** | **<.0001** |  |
| **Diversity** | **2** | **158** | **144.64289** | **<.0001** |  |
| **Removal Treatment** | **4** | **648** | **12.50147** | **<.0001** |  |
| **Diversity: Removal Treatment** | **8** | **648** | **8.03948** | **<.0001** |  |
|  |  |  |  |  |  |
| **Full Model: Rates of NDVI Senescence** |  |  |  |  |  |
|  |  |  |  |  |  |
| **Parameter** | **Estimate** | **Standard Error** | **DF** | **t** | **p** |
| (Intercept) | -0.00067 | 0.00053 | 648 | -1.27 | 0.2000 |
| **Diversity 4** | **-0.00235** | **0.00027** | **158** | **-8.78** | **<0.0001** |
| **Diversity 16** | **-0.00389** | **0.00027** | **158** | **-14.55** | **<0.0001** |
| **Soil Fungicide** | **0.0003** | **0.00012** | **648** | **2.47** | **0.0140** |
| Insecticide | 0.00022 | 0.00012 | 648 | 1.81 | 0.0710 |
| Foliar Fungicide | 0.00014 | 0.00012 | 648 | 1.19 | 0.2400 |
| All Pesticides | 0.00019 | 0.00012 | 648 | 1.56 | 0.1200 |
| **Diversity 4: Soil Fungicide** | **-0.00046** | **0.0002** | **648** | **-2.35** | **0.0190** |
| **Diversity 16: Soil Fungicide** | **-0.00047** | **0.0002** | **648** | **-2.42** | **0.0160** |
| **Diversity 4: Insecticide** | **-0.00046** | **0.0002** | **648** | **-2.36** | **0.0190** |
| Diversity 16: Insecticide | -0.00021 | 0.0002 | 648 | -1.08 | 0.2800 |
| **Diversity 4: Foliar Fungicide** | **0.00065** | **0.0002** | **648** | **3.35** | **0.0009** |
| Diversity 16: Foliar Fungicide | 0.00024 | 0.0002 | 648 | 1.23 | 0.2200 |
| **Diversity 4: All Pesticides** | **0.00056** | **0.0002** | **648** | **2.87** | **0.0043** |
| Diversity 16: All Pesticides | 0.00022 | 0.0002 | 648 | 1.1 | 0.2700 |

**Table S7.** Seasonal timing of maximum NDVI response to consumer removal treatments (soil fungicide, insecticide, foliar fungicide, and all pesticides combined) across a gradient of plant species richness (1, 4, and 16 species). Timing of maximum NDVI is defined as the Julian day of year where the maximum NDVI value appears within a given year for each experimental plot. This table is linked with Figure 5 in the main text.

$$Day of maximum NDVI \sim Diversity*Removal Treatment,$$

$$random=\sim1 | Year/Plot/Subplot$$

| **Source** | **numDF** | **denDF** | **F-value** | **p-value** |  |
| --- | --- | --- | --- | --- | --- |
| **(Intercept)** | **1** | **1044** | **5674.961** | **<.0001** |  |
| **Diversity** | **2** | **254** | **42.672** | **<.0001** |  |
| **Removal Treatment** | **4** | **1044** | **5.491** | **0.0002** |  |
| Diversity: Removal Treatment | 8 | 1044 | 0.904 | 0.5124 |  |
|  |  |  |  |  |  |
| **Full Model:**  **Timing of maximum NDVI** |  |  |  |  |  |
|  |  |  |  |  |  |
| **Parameter** | **Estimate** | **Standard Error** | **DF** | **t** | **p** |
| **(Intercept)** | **200.17** | **3.2** | **1044** | **62.79** | **<0.0001** |
| **Diversity 4** | **-12.99** | **3.7** | **254** | **-3.51** | **0.0005** |
| **Diversity 16** | **-23.45** | **3.7** | **254** | **-6.33** | **<0.0001** |
| Soil Fungicide | -0.44 | 2.2 | 1044 | -0.2 | 0.8400 |
| Insecticide | 1.6 | 2.2 | 1044 | 0.72 | 0.4700 |
| Foliar Fungicide | 4.02 | 2.2 | 1044 | 1.8 | 0.0730 |
| **All Pesticides** | **5.29** | **2.2** | **1044** | **2.37** | **0.0180** |
| Diversity 4: Soil Fungicide | -3.92 | 3.7 | 1044 | -1.07 | 0.2800 |
| Diversity 16: Soil Fungicide | -2.68 | 3.7 | 1044 | -0.74 | 0.4600 |
| Diversity 4: Insecticide | -1.98 | 3.7 | 1044 | -0.54 | 0.5900 |
| Diversity 16: Insecticide | -2.85 | 3.7 | 1044 | -0.78 | 0.4400 |
| Diversity 4: Foliar Fungicide | 2.73 | 3.7 | 1044 | 0.75 | 0.4500 |
| Diversity 16: Foliar Fungicide | -4.39 | 3.7 | 1044 | -1.2 | 0.2300 |
| Diversity 4: All Pesticides | -1.5 | 3.7 | 1044 | -0.41 | 0.6800 |
| Diversity 16: All Pesticides | -5.0 | 3.7 | 1044 | -1.37 | 0.1700 |

**Supplemental Figures.**


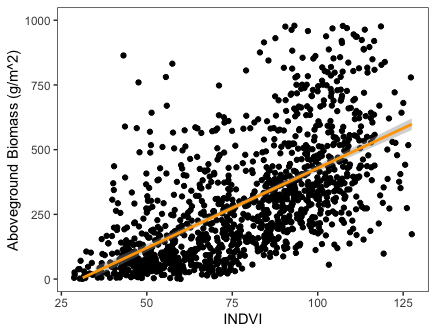


**Figure S1.** Integrated Normalized Difference Vegetation Index (INDVI) for the entire growing season generally correlates with aboveground biomass regardless of diversity or consumer removal treatment (R^2^=0.36, p < 0.0001).

**Figure S2.** Day of peak NDVI and NDVI time series curves are highly variable among plant species. Trend lines show local polynomial regression fit of each experimental treatment with an alpha parameter of 0.75. DOY = Julian day of year. Species abbreviations are defined as follows:

Achmi = *Achillea millefolium,* Agrsm = *Agropyron smithii,* Amoca = *Amorpha canescens,* Andge = *Andropogon gerardi,* Asctu = *Asclepias tuberosa,* Elyca = *Elymus canadensis,*

Koecr = *Koeleria cristata,* Lesca = *Lespedeza capitata,* Liass = *Liatris asperca,*

Luppe = *Lopinus perennis,* Panvi = *Panicum virgatum,* Petpu = *Petalostemum purporeum,*

Poapr = *Poa pratensis,*  Schsc = *Schizachyrium scoparium,* Sornu = *Sorghastrum nutans*,

16 Species Plots = a random combination of species found in this experiment (see Methods section for more details and Table S1).
